# Efficacy and Safety of Very Short-Term Dual Antiplatelet Therapy After Drug-Eluting Stents Implantation for Acute Coronary Syndrome: A Systematic Review and Meta-Analysis of Randomized Clinical Trials

**DOI:** 10.3389/fcvm.2021.660360

**Published:** 2021-09-07

**Authors:** Peng-Yu Zhong, Yao-Sheng Shang, Nan Bai, Ying Ma, Ying Niu, Zhi-Lu Wang

**Affiliations:** ^1^The First Clinical Medical College of Lanzhou University, Lanzhou, China; ^2^Department of Cardiology, The First Hospital of Lanzhou University, Lanzhou, China

**Keywords:** acute coronary syndrome, percutaneous coronary intervention, drug-eluting stents, dual antiplatelet therapy, trial sequential analysis

## Abstract

**Background and Objective:** Dual antiplatelet therapy (DAPT) is the basis for preventing ischemic events after percutaneous coronary intervention (PCI), and DAPT for 12 months has been the standard strategy recommended by the guidelines. However, patients with acute coronary syndrome (ACS) have a higher risk of thrombosis, and the application of very short-term DAPT (1–3 months) in patients with ACS is consistently controversial. The purpose of this study is to explore the efficacy and safety of DAPT for 1–3 months in patients with ACS who were implanted with drug-eluting stents (DES).

**Methods:** We conducted a systematic review and meta-analysis of randomized controlled trials that compared the very short-term (3 months or less) with long-term (12 months or more) DAPT in patients with ACS after PCI. The randomized controlled trials were included by searching PubMed, EMBASE, and Cochrane Library database. The relative risk (RR) and 95% CIs for endpoint events were calculated by the fixed effects model, and trial sequential analysis was applied to calculate the anticipated sample size and assess the results.

**Result:** A total of eight randomized controlled trials with 16,492 patients who met the inclusion criteria were conducted. There were no significant statistic differences in myocardial infarction (RR 1.05, 0.82–1.35, *P* = 0.68), stents thrombosis (RR 1.32, 0.85–2.07, *P* = 0.22), all-cause death (RR 0.87, 0.66–1.13, *P* = 0.29), and target vessel revascularization (RR 0.93, 0.76–1.13, *P* = 0.47). However, there were significant differences in major bleeding (RR 0.60, 0.50–0.73, *P* < 0.00001) and the net adverse cardiac and cerebrovascular events (RR 0.84, 0.74–0.95, *P* = 0.007).

**Conclusions:** The strategy of DAPT for 1–3 months not only has a significant effect in patients with ACS who were implanted with DES but also reduces the risk of major bleeding. The scheme of short-term DAPT followed by P2Y_12_ receptor inhibitor monotherapy is especially beneficial for patients with ACS. The results of this systematic review and meta-analysis are based on the application of new generation DES and new oral antiplatelet drugs in patients with ACS, which are difficult to use in the general population (Registered by PROSPERO, CRD 42020210520).

**Systematic Review Registration:**https://www.crd.york.ac.uk/PROSPERO/, identifier: CRD 42020210520.

## Introduction

Following the emergence of percutaneous coronary intervention (PCI), dual antiplatelet therapy (DAPT), including aspirin and P2Y_12_ receptor inhibitor, has gradually reduced ischemic events (1). The antiplatelet strategy has been continuously upgrading, and the strategy of ticagrelor combined with aspirin was recommended as class I in patients with the acute coronary syndrome (ACS) (2). ACS refers to a series of coronary artery diseases, including unstable angina, non-ST elevated myocardial infarction, and ST-elevated myocardial infarction. In addition, a series of clinical trials have confirmed that DAPT for at least 1 year can significantly reduce the ischemic events, and it was recommended by the 2012 ACCF/AHA/SCAI guidelines as a standard strategy in patients undergoing drug-eluting stents (DES) implantation (3–5). However, with the development of antiplatelet drugs, stent materials, and intravascular imaging, the risk of ischemia decreased after PCI. Therefore, a number of randomized controlled trials were designed to explore the deescalation of DPAT, which include reducing the dose of drugs, decreasing the duration of DAPT, and adjusting the match of DAPT (6–9).

In recent years, DAPT for 1–3 months followed by P2Y_12_ receptor inhibitor monotherapy has become a novel short-term DAPT strategy. Three trials have confirmed that this strategy was non-inferior to 12-month DAPT for the general population (6–8). Meanwhile, a network meta-analysis also showed that DAPT for 1–3 months followed by P2Y_12_ receptor inhibitor monotherapy is the best strategy compared with the other three DAPT strategies for general population (10). However, there still are controversies on the application of short-term DAPT for patients with ACS. Although two trials verified that shortening DAPT duration could reduce the risk of major bleeding for patients with ACS (11, 12), it could also result in a higher incidence of ischemic events than long-term DAPT (13).

Therefore, it is uncertain whether the short-term DAPT is a feasible strategy in ACS patients with implanted DES. This systematic review and meta-analysis was conducted to verify the efficacy and safety of very short-term (1–3 months) DAPT for patients with ACS who were with implanted DES, by comparing with long-term (≥12 months) DAPT.

## Methods

### Data Source and Quality Assessment

This systematic review and meta-analysis of randomized controlled trials were performed according to the preferred reporting items for systematic review and meta-analysis (PRISMA) guideline (14). PubMed, EMBASE, and Cochrane Library database were searched from inception to August 3, 2020. The search strategy of PubMed was as follows: “percutaneous coronary intervention” or “drug-eluting stent,” and “dual anti-platelet therapy” or “aspirin” or “clopidogrel” or “prasugrel” or “ticagrelor,” or “P2Y_12_ inhibitor” and “randomized controlled trial” with no language restrictions; the whole search strategies are shown in the Supplementary Appendix (Supplementary Tables 1–3). An update reminder for PubMed was created to keep up with the latest research. The inclusion criteria of the study met the following requirements: (a) randomized controlled trial (or subgroup analysis of a randomized controlled trial) that compared the very short-term DAPT (1–3 months) with long-term DAPT (≥12 months) in patients with ACS after PCI; (b) follow-up duration ≥12 months after the index PCI; and (c) reported incidence of the primary efficacy and/or safety outcomes that we want to explore. The exclusion criteria included non-randomized controlled trial and studies that did not report the data of patients with ACS. Two investigators (Peng-yu Zhong and Yao-sheng Shang) independently screened all titles, abstracts, and full-text articles of relevant studies, and then the trial eligibility was assessed following the inclusion and exclusion criterion. The disagreement was discussed to resolve by a third party (Nan Bai, Ying Ma, and Ying Niu). The Cochrane tool of Collaboration was used to assess the risk of bias for each trial, and the grades of recommendations assessment, development, and evaluation (GRADE) were conducted to evaluate the quality of each outcome (15, 16). The clinical protocols of all included trials were approved by local ethics, and informed consent of patients was obtained. The study protocol was registered in PROSPERO (CRD 42020210520).

### Data Acquisition and Clinical Outcomes

The baseline characteristics of studies and patients were extracted by two researchers independently, and the discrepancy was resolved through negotiation (Zhilu Wang). The primary efficacy outcomes included myocardial infraction, stent thrombosis, all-cause mortality, and target vessel revascualization. The safety outcome was major bleeding, and net adverse cardiac and cardiovascular events (NACCE) were evaluated as a composite outcome, which was defined as a composite of all-cause mortality, myocardial infraction, stroke, or major bleeding. The definition of outcomes in each trial is showed (Supplementary Table 4).

### Statistical Analysis

Review manager 5.4 and Stata 14.1 were adopted in this systematic review and meta-analysis. The Cochrane Q statistic with Pearson chi-square test and the Higgins *I*^2^ test was used to examine heterogeneity in the Review manager. Labbe and Galbraith plots were employed to further test heterogeneity in Stata. The relative risk (RR) as effect size was calculated by the fixed effects mode. Sensitivity analysis was performed to seek for the reason of heterogeneity by the “one-study-removed” method. Subgroup analyses and test for interaction were executed according to the different drugs of monotherapy after short-term DAPT. In addition, the Egger's and Bgger's test, and also the visual inspection of funnel plots, were hired to assess publication bias. Trial sequential analysis (TSA) version 0.9.5.10 software (Copenhagen Trial Unit, CTU) was applied to calculate the sample size and assess the results. The “Low-bias Based” option was selected to estimate the anticipated intervention effect according to the TAS manual (available from www.ctu.dk/tsa).

## Results

### Search Results and Study Characteristics

**Figure 1:**
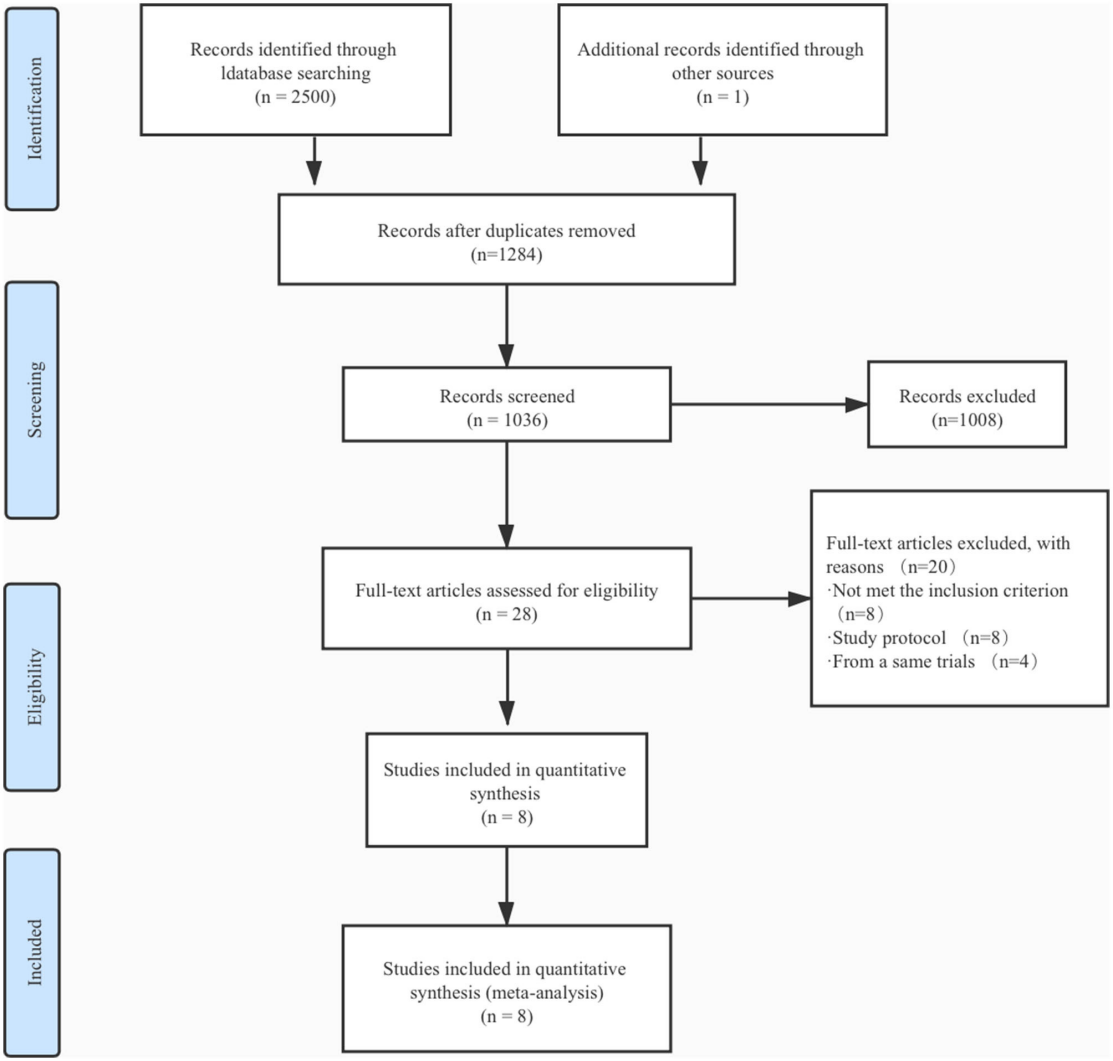
Flow diagram of literature search.

The process of literature screening and study selection is shown (Figure 1). A total of 2,500 articles were searched from PubMed, EMBASE, and Cochrane library database. Another article came from the 2019 American Heart Association annual scientific sessions, and the final manuscripts were not published (9). Finally, eight randomized controlled trials met the inclusion and exclusion criteria out of 28 full-text articles reviewed (6–11, 17, 18).

The characteristics of the studies included are showed (Table 1). A total of 21,138 patients were divided into short-term DAPT group (10,531) and long-term DAPT group (10,608). Of these eight trials, two trials exclusively enrolled patients with ACS and the other six trials included patients with ACS and chronic coronary syndrome, and the outcomes of the subgroup for patients with ACS were reported. The majority of studies were open-label and non-inferiority trials that used clopidogrel as the second antiplatelet agent, only one trial was a double-blind trial (9). In addition, five trials applied the strategy of short-term DAPT followed by P2Y_12_ receptor inhibitor monotherapy, and the rest of the trials used aspirin monotherapy after short-term DAPT. The type of DES was different in all studies, part of the patients in the RESET trial were implanted with the first-generation DES. Furthermore, patients in the other seven randomized controlled trials were implanted with second-generation DES or new-generation DES, the latter included new second-generation DES and bioresorbable polymer DES.

**Table 1:** Baseline characteristics of the included trials.

**Study**	**Reset**	**Optimize**	**Global leaders**	**Reduce**	**Stop DAPT2**	**Smart-choice**	**Twilight**	**TICO**
Authors	Byeong-Keuk Kim	Fausto Feres	Pascal Vranckx	Giuseppe De Luca	Hirotoshi Watanabe	Joo-Yong Hahn	Usman Baber	Byeong-Keuk Kim
Publication year	2012	2013	2018	2019	2019	2019	2019	2020
Study country	Korea	Brazil	18 Countries	Europe and Asia	Japan	Korea	11 Countries	Korea
Study cohort	ACS and CCS	ACS and CCS	ACS and CCS	ACS	ACS and CCS	ACS and CCS	ACS and CCS	ACS
Comparison	3 vs. 12	3 vs. 12	1 vs. 12	3 vs. 12	1 vs. 12	3 vs. 12	3 vs. 15	3 vs. 12
Study total size	2,217	3,119	15,968	1,496	3,045	2,993	9,006	3,056
ACS cohort size	601	996	7,487	1,496	1,148	1,741	4,614	3,056
Short-term DAPT (n)	301	494	3,750	751	565	870	2,273	1,527
Long-term DAPT (*n*)	300	502	3,737	745	583	871	2,341	1,529
Stent type	ZES:EES:SES	ZES	Biolimus A9-eluting stent	COMBO	CoCr-EES	CoCr-EES PtCr-EES BP-SES	Locally approved DES	BP-SES
Follow up (months)	12	12	24	24	12	12	15	12

*ZES, zotarolimus-eluting stent; EES, everolimus-eluting stent; SES, sirolimus-eluting stent; CoCr-EES, cobalt chromium everolimus-eluting stent; PtCr-EES, platinum chromium everolimus-eluting stent; BP-SES, bioresorbable polymer sirolimus-eluting stent; COMBO, Combines a CD34 + antibody and sirolimus (OrbusNeich, Fort Lauderdale, Florida)*.

The baseline characteristics of patients are shown (Table 2). The baseline characteristics of age, sex, hypertension, and diabetes were similar in each trial. However, there was heterogeneity in the incidence of ST-elevation myocardial infarction in the included trials. The incidence of ST-elevation myocardial infarction was high in REDUCE trial (49.3% in the short-term DAPT group and 45.2% in the long-term DAPT group). However, TWILIGHT and OPTIMIZE trials only included patients with non-ST-elevation myocardial infarction and unstable angina. In addition, the incidence of previous myocardial infraction was similar (13.8–25.4% in the short-term DAPT and 13.2–25.2% in the long-term DAPT) in the ACS subgroup of TWILIGHT, OPTIMIZE, STOP DAPT-2, and GLOBAL LEADERS trials, but the proportion of others was <4.3%.

**Table 2:** Baseline characteristics of the patients included.

**Study**	**Reset**	**Optimize**	**Global leaders**	**Reduce**	**Stop DAPT-2**	**Smart-choice**	**Twilight**	**TICO**
STEMI (%)	AMI 51.8/48.7	0/0	28.3/27.6	49.3/45.2	19.4/17.9^a^	11.0/10.0^a^	0/0	35.6/36.4
NSTEMI (%)		17.0/16.7b	44.9/45.2	35.6/41.0	5.4/6.6^a^	16.0/15.4^a^	–	35.3/31.9
UAP (%)	48.2/51.3	83.0/83.3	26.8/27.2	15.2/13.8	12.9/14.2^a^	31.2/32.8^a^	–	29.1/31.7
Age (years)	62.4/62.4^a^	61.3/61.9^a^	>75 (14.9/14.7%)	61/60	68.1/69.1^a^	64.6/64.4^a^	64.2/64.2	61/61
Men (%)	64.4/62.9^a^	63.5/63.1^a^	76.8/77.1	82.6/77.3	78.9/76.5^a^	72.7/74.2^a^	74.5/75.2	78.8/80.1
Hypertension (%)	62.3/61.4^a^	86.4/88.2^a^	68.6/67.9	50.7/50.7	73.7/74.0^a^	61.6/61.3^a^	–	49.8/51.1
Diabetes (%)	29.8/28.8^a^	35.4/35.3^a^	21.6/21.3	21.7/19.6	39.0/38.0^a^	38.2/36.8^a^	35.6/34.3	27.4/27.3
Hyperlipidemia (%)	57.7/59.9^a^	63.2/63.7^a^	60.8/62.0	46.3/44.9	74.4/74.8^a^	45.1/45.5^a^	–	–
Current smoker (%)	25.2/22.8^a^	18.6/17.3^a^	34.3/33.6	42.1/42.7	26.6/20.6^a^	28.4/24.5^a^	23.3/26.6	36.3/38.4
Previous MI (%)	1.8/1.6^a^	24.4/23.1^a^	18.3/18.6	–	13.8/13.2^a^	4.1/4.3^a^	25.4/25.2	4.2/3.2
Previous PCI (%)	3.5/3.0^a^	20.9/19.1^a^	22.8/23.4	11.7/9.8	33.5/35.1^a^	Previous revascularization 11.5/11.8^a^	34.2/34.4	8.8/8.3
Previous CABG (%)	0.2/0.6^a^	7.1/8.2^a^	3.5/3.9	2.8/2.8	1.1/2.8^a^		8.8/8.5	0.52/0.65
LVEF (%)	64.2/63.9^a^	<50%(36.1/35.4^a^)	–	–	59.8/59.7^a^	60.0/59.9^a^	–	–
Number of stents (n)	–	1.6/1.6^a^	–	>2 17.4/18.1	1.3/1.3^a^	–	–	1.37/1.37
Total stent length (mm)	–	32.8/32.7^a^	–	23.0/23.0	30.3/30.5^a^	38.0/37.8^a^	40.5/39.8	35/35

*Data are shown as groups with short-term/long-term DAPT.*

*AMI, acute myocardial infarction; CABG, coronary artery bypass grafting; DAPT, dual antiplatelet therapy; LVEF, left ventricular ejection fraction; MI, myocardial infarction; NSTE-ACS, non-ST-elevation acute coronary syndrome; NSTEMI, non-ST-elevation myocardial infarction; PCI, percutaneous coronary intervention; STEMI, ST-elevation myocardial infarction; UAP, unstable angina.*

a *Data of all cohort including stable coronary artery disease*.

### The Primary Efficacy Outcomes

**Figure 2:**
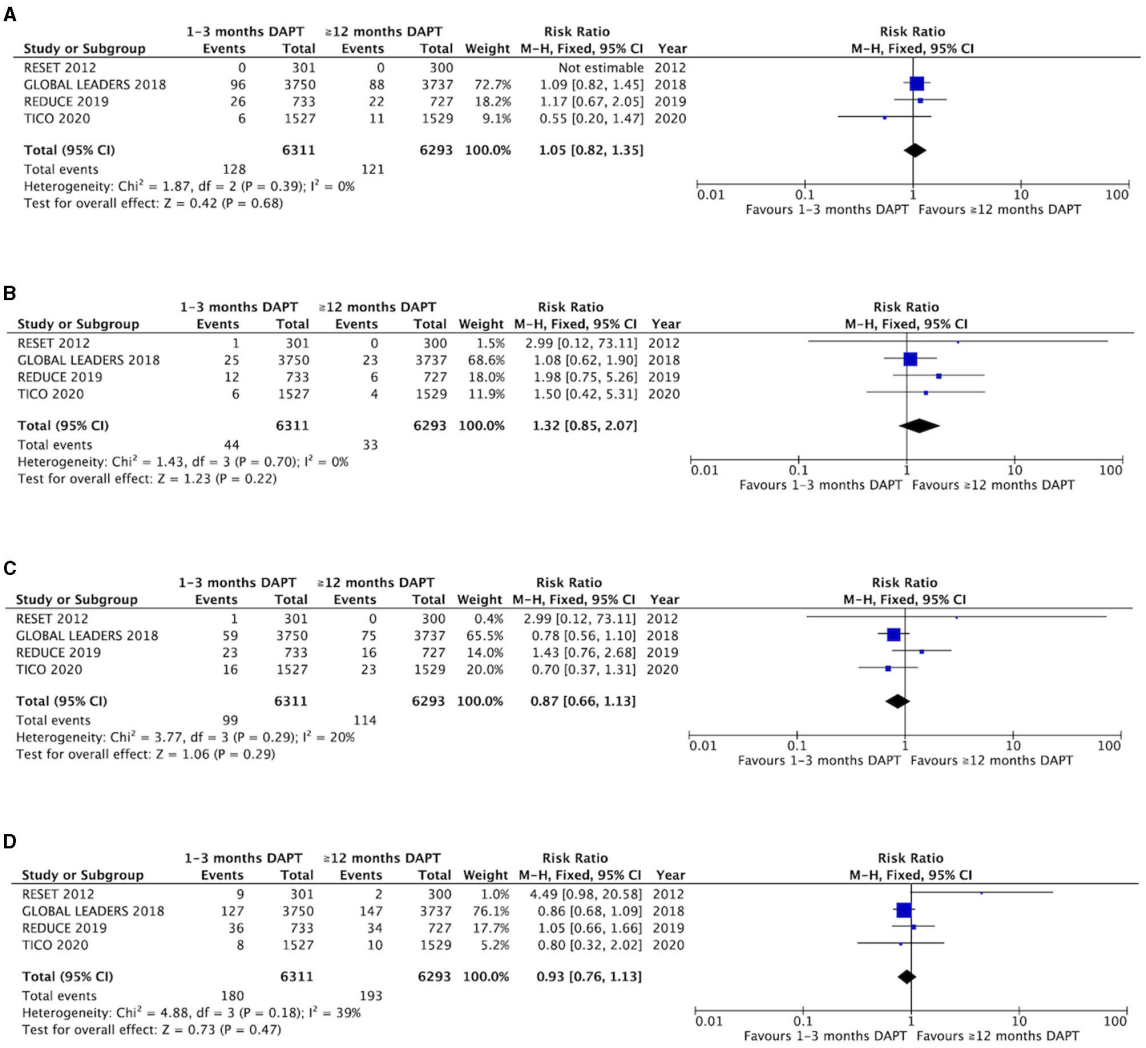
Comparison of efficacy endpoints between groups with short-term and long-term DAPT. **(A)** myocardial infarction, **(B)** stent thrombosis, **(C)** all-cause death, **(D)** target vessel revascualization.

The myocardial infarction and stent thrombosis are reported in the four trials (Figures 2A,B). There were no significant differences and heterogeneity in myocardial infarction between groups with short-term and long-term DAPT (2.0 vs. 1.9%, RR 1.05, 0.82–1.35, *P* = 0.68, *I*^2^ = 0%, *P*_Heterogeneity_ = 0.39). The difference and heterogeneity of stent thrombosis was similar to myocardial infarction between the two groups (0.70 vs. 0.52%, RR 1.32, 0.85–2.07, *P* = 0.22, *I*^2^ = 0%, *P*_Heterogeneity_ = 0.70). The results of subgroup analysis are shown in Supplementary Figures 1, 2, and there were no significant differences between P2Y_12_ inhibitor monotherapy and aspirin monotherapy after short-term DAPT.

The all-cause mortality is also presented in the four trials (Figure 2C). No significant difference was not found between the groups with 1–3 months DAPT and ≥12 months DAPT (1.6 vs 1.8%, RR 0.87, 0.66–1.13, *P* = 0.29, *I*^2^ = 20%, *P*_Heterogeneity_ = 0.18). In addition, four trials mentioned the outcome of target vessel revascularization, which shows that there was no significant difference and only a mild heterogeneity between the two groups (2.9 vs. 3.1%, RR 0.93, 0.76–1.13, *P* = 0.47, *I*^2^ = 39%, *P*_Heterogeneity_ = 0.18) (Figure 2D). One study that produced heterogeneity was identified by sensitivity analysis, and the heterogeneity of the target vessel revascularization was reduced after excluding the results of this trial (*I*^2^ = 0%, *P*_Heterogeneity_ = 0.73), and there is no significant difference between the two groups (1.6 vs. 1.8%, RR 0.89, 0.73–1.09, *P* = 0.27) (Supplementary Figure 3). The results of subgroup analysis are shown (Supplementary Figures 4, 5). No significant differences were found between P2Y_12_ inhibitor monotherapy and aspirin monotherapy after short-term DAPT.

### The Primary Safety Outcomes and Subgroup Analysis

**Figure 3:**
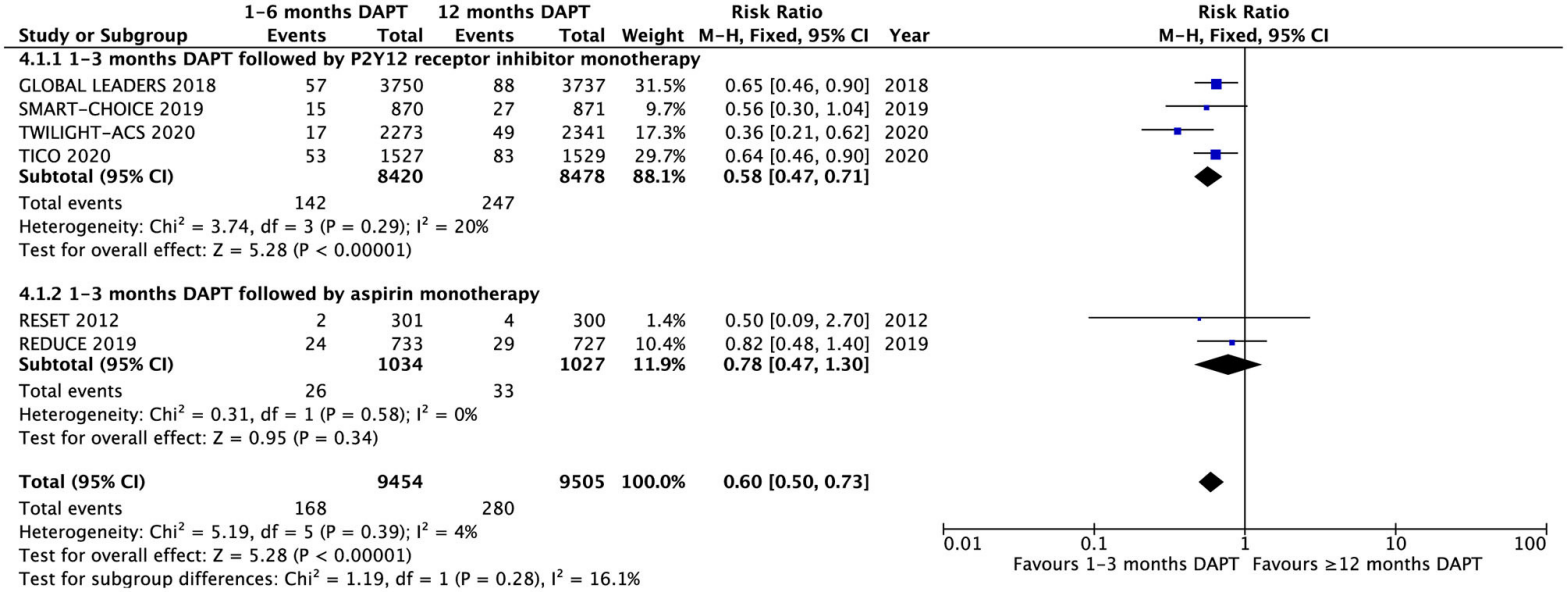
Subgroup analysis of major bleeding between groups with short-term and long-term DAPT.

The major bleeding is showed in the six trials (Figure 3). There was significant difference in major bleeding between the groups with 1–3 months DAPT and ≥12 months DAPT (1.8 vs. 2.9%, RR 0.60, 0.50–0.73, *P* < 0.00001, *I*^2^ = 4%, *P*_Heterogeneity_ = 0.39). The subgroup analysis was conducted according to the difference in the antiplatelet strategy, which showed that there was a significant difference in the strategy of short-term DAPT followed by P2Y_12_ receptor inhibitor monotherapy (1.7 vs. 2.9%, RR 0.58, 0.47–0.71, *P* < 0.00001, *I*^2^ = 20%, *P*_Heterogeneity_ = 0.29). In contrast, there was no significant difference in the strategy of short-term DAPT followed by aspirin monotherapy (2.5 vs. 3.2%, RR 0.78, 0.47–1.30, *P* = 0.34, *I*^2^ = 0%, *P*_Heterogeneity_ = 0.58). Meanwhile, there are differences between the two short-term strategies, but no statistical significance (*I*^2^ = 16.1%, *P*_interaction_ = 0.28) (Figure 3).

### The Composite Endpoint and Subgroup Analysis

**Figure 4:**
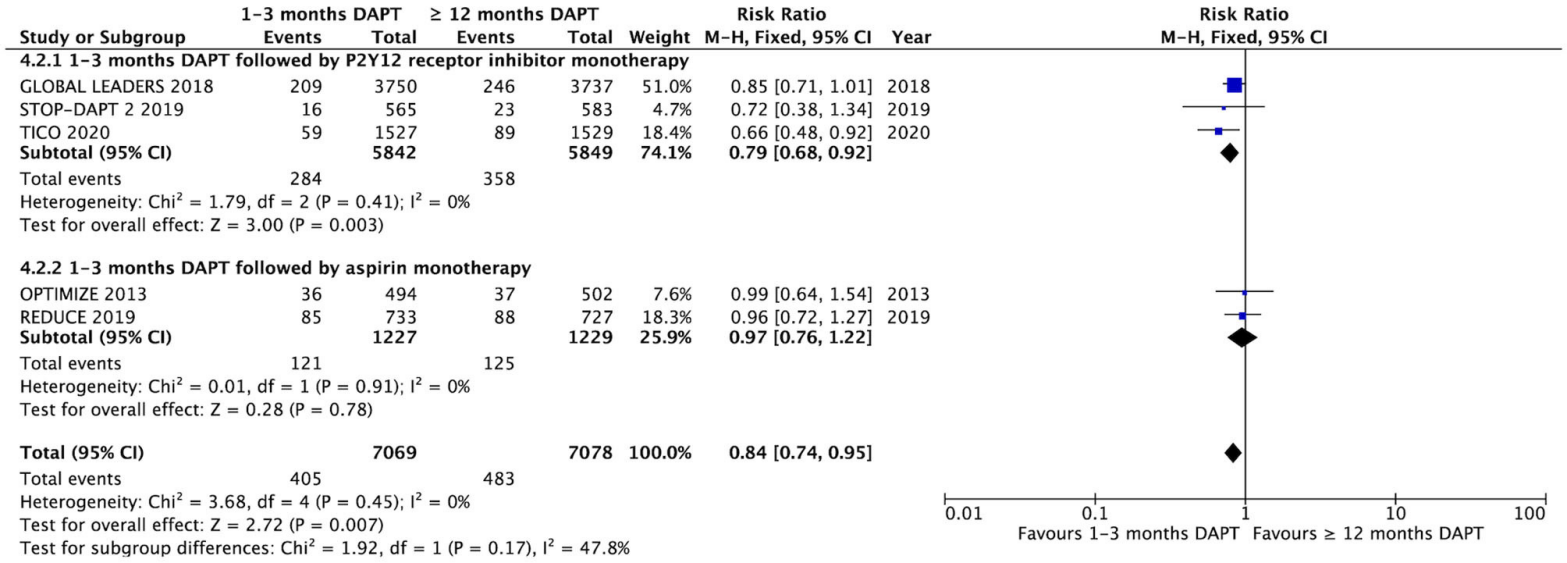
Comparison of complication endpoints of NACCE and subgroup analysis between groups with short-term and long-term DAPT.

The NACCE is reported in five of the eight trials (Figure 4). There was a significant difference in NACCE between the groups of patients with ACS with 1–3 months DAPT and ≥12 months DAPT (5.0 vs. 5.7%, RR 0.88, 0.77–1.01, *P* = 0.007, *I*^2^ = 0%, *P*_Heterogeneity_ = 0.87). According to the subgroup analysis, short-term DAPT followed by P2Y_12_ receptor inhibitor monotherapy can significantly reduce the incidence of NACCE compared with long-term DAPT. However, aspirin monotherapy after short-term DAPT did not reduce the incidence of NACCE compared with long-term DAPT (Figure 4).

### Trial Sequential Analysis, Assessment of Quality, and Publication Bias

The TSA is conducted in each outcome (Supplementary Figure 6). The curves of the primary efficacy outcomes were under the conventional boundary and did not meet the anticipated sample size. In addition, the curve of major bleeding was beyond the conventional boundary and met the expected sample size. The curve of NACCE was also beyond the TSA boundary but did not reach the sample size. The risk of bias is assessed (Supplementary Figure 7). The risk of selection, detection, and reporting bias were low in all trials, and a high risk of performance bias was found in seven of the eight trials. The quality assessments of GRADE evidence for each outcome were demonstrated (Supplementary Table 5). There was high evidence of major bleeding and NACCE in the outcomes, but moderate evidence of myocardial infarction, all-cause mortality, and target vessel revascularization. There was no publication bias in all outcomes. The outcomes of trials included are distributed symmetrically in funnel plot, and the *P*-value of the Begg's and Egger's are more than 0.05 in all outcomes (Supplementary Figure 8; Supplementary Table 6).

## Discussion

The finding of this meta-analysis indicates that the very short-term DAPT was not associated with a higher risk of myocardial infarction, stent thrombosis, all-cause death, and target vessel revascularization compared with long-term DAPT strategy. However, the short-term DAPT can significantly reduce the incidences of major bleeding and NACCE compared with long-term DAPT. In addition, the GRADE evidence levels of each safety and efficacy outcome were high and moderate, respectively, according to the certainty of the evidence.

Since the results of this meta-analysis are from particular situations and patients with ACS, the conclusions of this study need to be applied carefully. Firstly, more than 90% of the patients were implanted with new generation DES in this study, which has a thinner platform, absorbable polymer, and a more ideal antiproliferative drug compared with first- and second-generation DES (19). A network meta-analysis of 49 randomized controlled trials demonstrated that the biodegradable polymer DES could significantly reduce the stent thrombosis compared with the bare metal stent, namely the first-generation DES and some of second-generation DES (20). Therefore, new-generation DES provides the possibility and foundation for the application of very short-term DAPT. In addition, second-generation DES was widely applied in clinical practice, but it is not clear whether the new-generation DES is better than the second-generation DES. Although a meta-analysis has proved that there were no differences between the second-generation DES and the new-generation DES in the general population (21), the result in patients with ACS is uncertain. Secondly, according to subgroup analysis of major bleeding, the strategy of P2Y_12_ receptor inhibitor monotherapy after short-term DAPT significantly decreased major bleeding, while aspirin monotherapy after short-term DAPT could not reduce the risk of major bleeding. However, the difference between these two short-term strategies was not statistically significant. Therefore, the conclusion that P2Y_12_ receptor inhibitor monotherapy is better than aspirin monotherapy after 1–3 months DAPT is not enough, which is an indirect comparison by subgroup analysis. The HOST-EXAM trial is the first trial that directly compared these two short-term DAPT strategies, which showed that clopidogrel monotherapy after short-term DAPT can significantly reduce the incidence of the primary outcome (HR, 0.73 0.59–0.9) compared with aspirin monotherapy after short-term DAPT (22). However, whether ticagrelor or prasugrel monotherapy after short-term DAPT is also superior to aspirin monotherapy after short-term DAPT is uncertain. Therefore, it is necessary to study the difference in bleeding risk between aspirin and P2Y_12_ receptor inhibitors, which needs to be directly compared in the further randomized controlled trial (23). Finally, other factors need to be considered in clinical practice. In general, the short-term DAPT is suitable for patients with a high risk of bleeding. A randomized controlled trial published at the 2020 conference of Transcatheter Cardiovascular Therapeutics confirmed that aspirin monotherapy after 1–3 months of DAPT is safer than 12 months of DAPT for patients with a high risk of bleeding (24). Meanwhile, the ethnic characteristics of platelet reactivity are important factors affecting the antiplatelet effect. The platelet reactivity from East Asians is higher than that of Westerners. That is to say, the risk of bleeding is high and the risk of ischemic events is low in East Asians (25). Therefore, the characteristics of different races for patients after PCI should be sufficiently considered, and it was appropriate to shorten DAPT duration for East Asian patients (26). In addition, the duration of P2Y_12_ receptor inhibitor monotherapy after short-term DAPT is uncertain. Compared with aspirin, prolonging P2Y_12_ receptor inhibitor monotherapy will bring more economic pressures, more drug intolerance, and lower compliance.

All studies in this meta-analysis were randomized controlled trials, and the risk of bias was assessed by the Cochrane tool of Collaboration. The results showed that there was a low risk of bias in selection, detection, and reporting bias, but a high risk of bias in performance, because seven of the eight trials did not blind participants and personnel. According to the results of this study, although the efficacy of short-term and long-term DAPT is similar, there is a significant difference in the incidences of major bleeding and NACCE, and the risk is reduced by 40 and 16%, respectively. Meanwhile, the risk of major bleeding is reduced by 46% in the strategy of short-term DAPT followed by P2Y_12_ receptor inhibitor monotherapy based on the subsequent subgroup analysis. However, there is no significant difference between the short-term DAPT followed by aspirin monotherapy and the long-term DAPT. Four of the eight randomized controlled trials applied P2Y_12_ receptor inhibitor as monotherapy. Each trial reduced major bleeding with a consistency and a no dose-response relationship was found because there was a uniform drugs dose in the main included trials followed by single aspirin (75–100 mg per day), single clopidogrel (75 mg per day), and two ticagrelors (90 mg per day). In addition, the sparsity data and repeated significance tests are important reasons for the increased risk of random errors in meta-analysis (27), and the TSA was conducted in this study for all outcomes to assess this risk. According to the results of TSA, the curve of major bleeding exceeded the conventional boundary and met the expected sample size. In short, the conclusion that the short-term DAPT can reduce the risk of bleeding in ACS patients after PCI should be considered as a true positive trial, which does not need more randomized controlled trials to prove. Meanwhile, the conclusions of this meta-analysis are similar to the European Society of Cardiology 2020 guideline, which recommended that patients who met non-ST-segment elevation ACS and PERCISE-DAPT score ≥25 or ARC-HBR should be considered for 3-month DAPT followed by aspirin (28). Finally, PubMed, EMBASE, and Cochrane Library database were searched with no language limitation in this study. The detailed search strategy was supplied in supplementary, and the selection and inclusion of trials can be replaced. The small study effect is the main reason for the publication bias, but the whole trial was a large sample study, and no publication bias was found by the funnel plot and the statistic test.

A systematic review and meta-analysis by Naoki Misumida et al. compared the efficacy and safety of DAPT between 3–6 months and ≥12 months in patients with ACS (29), which demonstrated that there was no significant difference in myocardial infarction (OR 1.21, 0.94–1.57, *P* = 0.14), stent thrombosis (OR 1.54, 1.00–2.38, *P* = 0.052), and major bleeding events (OR 0.74, 0.49–1.11, *P* = 0.14). Similarly, there was no significant difference in efficacy between DAPT for 1–3 months and long-term DAPT in this meta-analysis. More importantly, the DAPT for 1–3 months significantly decreased the risk of major bleeding. However, the strategy of aspirin monotherapy after DAPT for 3–6 months was applied to the whole trial of Naoki Misumida et al. Therefore, the results of the two studies are consistent according to the subgroup analysis, and the shift from aspirin to Y2P_12_ receptor inhibitor monotherapy has clinical benefits for major bleeding.

## Limitations

This systematic review and a meta-analysis of randomized clinical trials may have some limitations. First, there are some differences in the baseline characteristics of randomized controlled trials included. The double blinding design was only implemented in one trial, and other trials were open-label trials. Second, the different incidence of ST-elevation myocardial infarction in each trial may be an important factor of heterogeneity, which was more than 45% in both RESET and REDUCE trials (12, 17). However, TWILIGHT and OPTIMIZE trials did not include the patients with ST-elevation myocardial infarction (9, 18). Therefore, although only patients with ACS were included in the study, the low risk of ischemia or complex lesion limited the application of short-term DAPT in all patients with ACS. Third, there were significant differences in all stents included in the trials. The first- and second-generation DES were implemented in the two trials published in 2012 and 2013, respectively (17, 18), and patients used the new generation DES in other trials published in the past 2 years. Fourth, there are heterogeneities in the definition of major bleeding. BARC 3 or 5 is almost the same as TIMI minor or major, and four trials reported one major bleeding among them. However, REDUCE and SMART-CHOICE trials only reported the data of BARC 2, 3, or 5 in patients with ACS. Fifth, a result of this is a study-level meta-analysis. There is no sufficient data of each outcome in all trials, and the main data came from the ACS subgroup. Last, only the curve of NACCE surpassed the TSA boundary and the outcome of major bleeding reached the anticipate sample size, other outcomes did not. Therefore, a false negative result may be acquired and more randomized controlled trials are needed to meet the expected sample size, such as STOP DAPT-2-ACS (available from https://www.ClinicalTrials.gov/show/NCT03462498).

## Conclusions

This systematic review and meta-analysis demonstrates that the very short-term DAPT after PCI was not associated with increased risk of myocardial infarction, stent thrombosis, all-cause death and target vessel revascularization in patients with ACS compared with the long-term DAPT. Meanwhile, P2Y_12_ receptor inhibitor monotherapy after DAPT for 1–3 months can reduce the risk of bleeding and improve the clinical net benefit. However, the duration of P2Y_12_ receptor inhibitor monotherapy remains to be further explored, and how to choose aspirin and P2Y_12_ receptor inhibitor after short-term DAPT is still unknown.

## Data Availability Statement

The raw data supporting the conclusions of this article will be made available by the authors, without undue reservation.

## Author Contributions

PYZ: study design, data collection, data analysis, and manuscript. YSS: data collection, data analysis, and validation. NB, YM, and YN: data collection and validation. ZLW: scientific revision of the manuscript.

## Conflict of Interest

The authors declare that the research was conducted in the absence of any commercial or financial relationships that could be construed as a potential conflict of interest.

## Publisher's Note

All claims expressed in this article are solely those of the authors and do not necessarily represent those of their affiliated organizations, or those of the publisher, the editors and the reviewers. Any product that may be evaluated in this article, or claim that may be made by its manufacturer, is not guaranteed or endorsed by the publisher.
