# Supplementary material for: Efficacy and Safety of Very Short-Term Dual Antiplatelet Therapy After Drug-Eluting Stents Implantation for Acute Coronary Syndrome: A Systematic Review and Meta-Analysis of Randomized Clinical Trials
Source: Front Cardiovasc Med. 2021 Sep 7;8:660360. doi: 10.3389/fcvm.2021.660360 (PMC8452852; doi:10.3389/fcvm.2021.660360)
Supplement: Supplementary file 1 [file Data_Sheet_1.DOC]

**Supplemental Table 1. PubMed search strategy.**

| **#** | **Searches** | **Results** |
| --- | --- | --- |
| **#1** | "percutaneous coronary intervention"[Mesh] OR "coronary intervention*, percutaneous":[tiab] OR "intervention*, percutane  -ous coronary":[tiab] OR "pci":[tiab] OR "percutaneous coronary revascularization":[tiab] OR "coronary revascularization*, percu  -taneous":[tiab] OR "percutaneous coronary revascularizations":  [tiab] OR "revascularization*, percutaneous coronary":[tiab] OR "drug eluting stent*":[tiab] OR "des":[tiab] | **87881** |
| **#2** | "Dual Anti-Platelet Therapy"[Mesh] OR "dual antiplatelet therap  -y":[tiab] OR "dapt":[tiab] OR "aspirin":[tiab] OR "clopidogrel":  [tiab] OR "prasugrel":[tiab] OR "ticagrelor":[tiab] OR "p2y12 in  -hibitor":[tiab] OR "anti-platelet therapies, dual":[tiab] OR "anti-  platelet therapy, dual":[tiab] OR "dual anti-platelet therapy":[ti  -ab] OR "dual anti-platelet therapies":[tiab] | **59916** |
| **#3** | "Randomized Controlled Trial"[Publication Type] OR "controll  -ed clinical trial":[tiab] OR "clinical trials, randomized":[tiab] OR "trials, randomized clinical":[tiab] OR "randomized clinical studies":[tiab] | **517822** |
| **#4** | "Systematic Review"[Publication Type] OR "Meta-Analysis"  [Publication Type] OR "Review"[Publication Type] OR "meta-  analysis":[ti] OR "systematic review":[ti] OR "literature review"  :[ti] OR "expert consensus":[ti] OR "case report":[ti] | **3005415** |
| **#5** | **#1 AND #2 AND #3 NOT #4** | **976** |

**Supplemental Table 2. EMBASE search strategy.**

| **#** | **Searches** | **Results** |
| --- | --- | --- |
| **#1** | 'percutaneous coronary intervention':kw OR 'coronary inter  -vention*, percutaneous':ab,ti OR 'intervention*, percutaneous coronary':ab,ti OR 'pci':ab,ti OR 'percutaneous coronary revascularization*':ab,ti OR 'coronary revascularization*, percutaneous':ab,ti OR 'revascularization*, percutaneous coronary':ab,ti OR 'drug eluting stents':kw OR 'drug-eluting stent':ab,ti OR 'des':ab,ti | **123664** |
| **#2** | 'dual antiplatelet therapy'/exp OR 'dapt':ab,ti OR 'aspirin':ab,ti OR 'clopidogrel':ab,ti OR 'prasugrel':ab,ti OR 'ticagrelor':ab,ti OR 'p2y12 inhibitor':ab,ti OR 'anti-platelet therapies, dual':ab,  ti OR 'anti-platelet therapy, dual':ab,ti OR 'dual anti-platelet  therapy':ab,ti OR 'dual anti-platelet therapies':ab,ti | **96001** |
| **#3** | 'randomized controlled trial'/exp OR 'controlled clinical trial':ab,ti OR 'clinical trials, randomized':ab,ti OR 'trials, randomized clinical':ab,ti OR 'randomized clinical studies':  ab,ti | **621793** |
| **#4** | 'systematic review'/exp OR 'meta analysis'/exp OR 'review'  /exp OR 'meta-analysis':ti OR 'systematic review':ti OR 'literature review':ti OR 'expert consensus':ti OR 'case report'/exp OR 'case report':ti | **5354446** |
| **#5** | **#1 AND #2 AND #3 NOT #4** | **883** |

**Supplemental Table 3. Cochrane Library search strategy.**

| **#** | **Searches** | **Results** |
| --- | --- | --- |
| **#1** | MeSH descriptor percutaneous coronary intervention explode all trees OR (coronary intervention*, percutaneous):ti,ab,kw OR (intervention*, percutaneous coronary):ti,ab,kw OR (pci):ti,ab,kw OR (percutaneous coronary revascularization*):  ti,ab,kw OR (coronary revascularization*, percutaneous):ti,  ab,kw OR (revascularization*, percutaneous coronary):ti,ab,  kw OR (drug eluting stent*):ti,ab,kw OR (drug-eluting stent):  ti,ab,kw OR (des):ti,ab,kw | **17508** |
| **#2** | (dual antiplatelet therapy):ti,ab,kw OR (dapt):ti,ab,kw OR (aspirin):ti,ab,kw OR (clopidogrel):ti,ab,kw OR (prasugrel):ti,  ab,kw OR (ticagrelor):ti,ab,kw OR (p2y12 inhibitor):ti,ab,kw OR (anti-platelet therapies, dual):ti,ab,kw OR (anti-platelet therapy, dual):ti,ab,kw OR (dual anti-platelet therapy):ti,ab,  kw OR (dual anti-platelet therapies):ti,ab,kw | **18055** |
| **#3** | 'MeSH descriptor randomized controlled trial explode all trees OR (controlled clinical trial):ti,ab OR (clinical trials, randomized):ti,ab OR (trials, randomized clinical):ti,ab OR (randomized clinical studies):ti,ab | **184792** |
| **#4** | **#1 AND #2 AND #3** | **641** |

**Supplemental Table 4**. Definition of outcomes in each trial.

| Study name | Primary outcome | Stent thrombosis | Major bleeding |
| --- | --- | --- | --- |
| RESET ^[15]^ | Composite of cardiovascular death, MI, ST, target-vessel revascularization, or major bleeding | Definite or probable ST by ARC criteria | TIMI major or minor bleeding |
| OPTIMIZE ^[16]^ | Composite of all-cause death, MI, stroke, or major bleeding | Definite or probable ST by ARC criteria | Modified major REPLACE-2 and severe or life-threatening GUSTO criteria |
| GLOBAL LEADERS^[6]^ | Composite of all-cause death  or new Q-wave myocardial infarction | Definite or probable ST by ARC criteria | BARC type 3 or 5 bleeding |
| REDUCE ^[17]^ | Composite of all-cause death, MI, ST, stroke, target-vessel revascularization, or major bleeding | Definite or probable ST by ARC criteria | BARC 2,3,5 |
| STOP-DAPT 2^[7]^ | Composite of cardiovascular death, MI, ST, stroke, or TIMI major or  minor bleeding | Definite or probable ST by ARC criteria | TIMI major or  minor bleeding |
| SMART-CHOICE^[8]^ | Composite of all-cause  death, MI, or stroke | Definite or probable ST by ARC criteria | BARC 2-5 |
| TWILIGHT^[9]^ | BARC type 2, 3, or 5 bleeding | Definite or probable ST by ARC criteria | BARC type 3 or 5 bleeding |
| TICO^[10]^ | Composite of major bleeding, death, MI, ST, stroke, or target-vessel revascularization | Definite or probable ST by ARC criteria | TIMI major or minor bleeding |

**Abbreviations:** ARC; Academic Research Consortium, BARC; Bleeding Academic Research Consortium, GUSTO; Global Utilization of Streptokinase and Tissue Plasminogen Activator for Occluded Coronary Arteries, MI; myocardial infarction, REPLACE-2; Randomized Evaluation of PCI Linking Angiomax to Reduced Clinical Events, ST; stent thrombosis, TIMI; Thrombolysis in Myocardial Infarction

**Supplemental Table 5.** Quality assessments of GRADE evidence for each outcome.


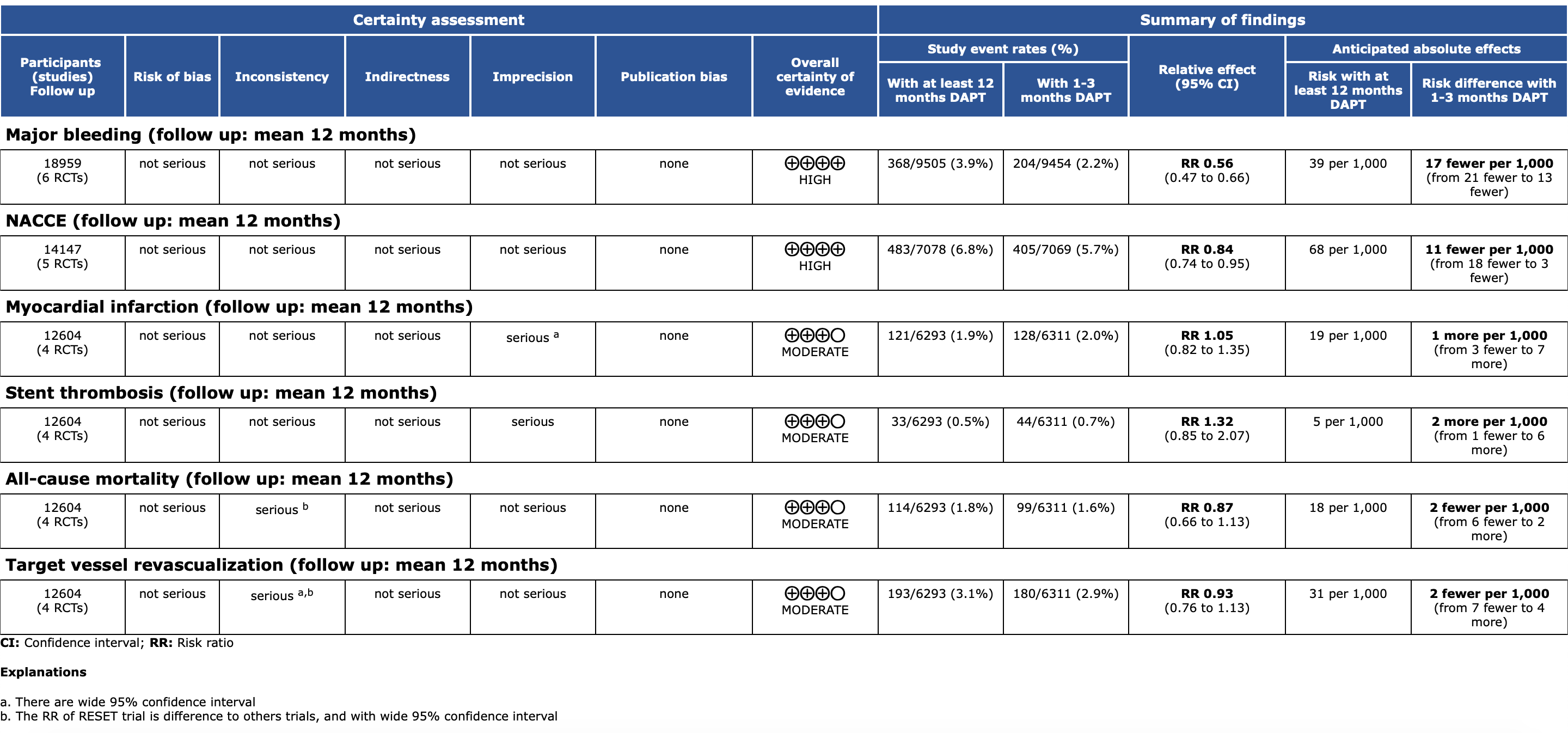


**Supplemental Table 6.** The Begg’s and Egger’s Test in STATA of each outcome.

| Endpoints | *P* (Begg’s test) | *P* (Eegg’s test) |
| --- | --- | --- |
| myocardial infarction | 1.000 | 0.493 |
| stent thrombosis | 0.734 | 0.224 |
| all-cause death | 0.734 | 0.440 |
| target vessel revascularization | 0.308 | 0.280 |
| Major bleeding | 1.000 | 0.509 |
| MACCE | 1.000 | 0.908 |
| NACCE | 0.806 | 0.819 |


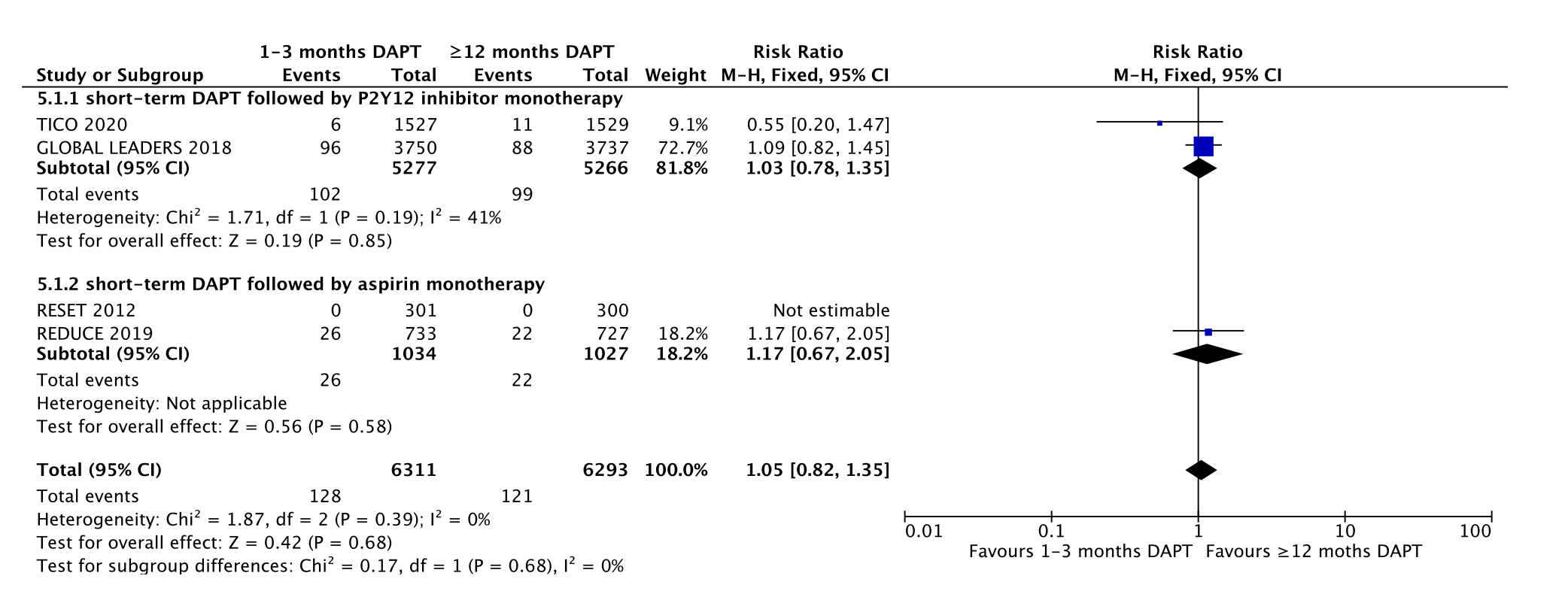


**Supplemental Figure S1.** The forest plot of subgroup analysis for myocardial infraction.


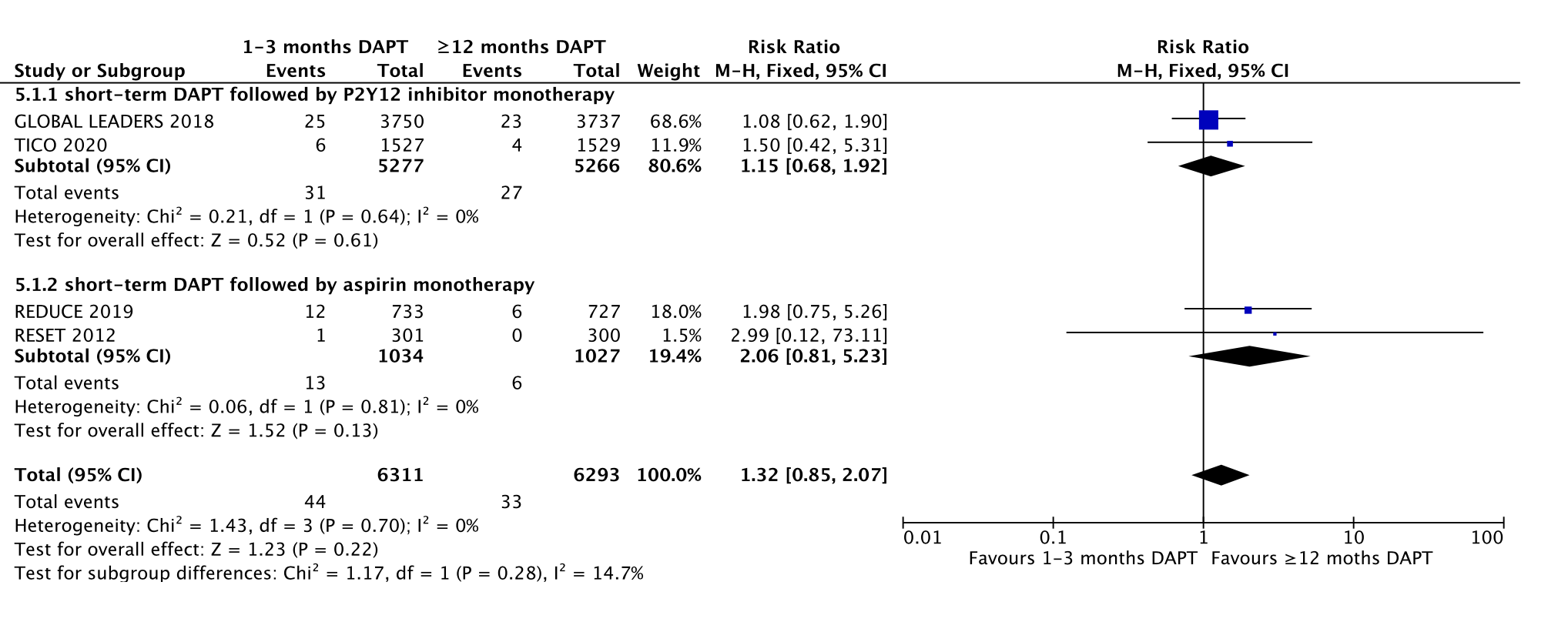


**Supplemental Figure S2.** The forest plot of subgroup analysis for stent thrombosis.


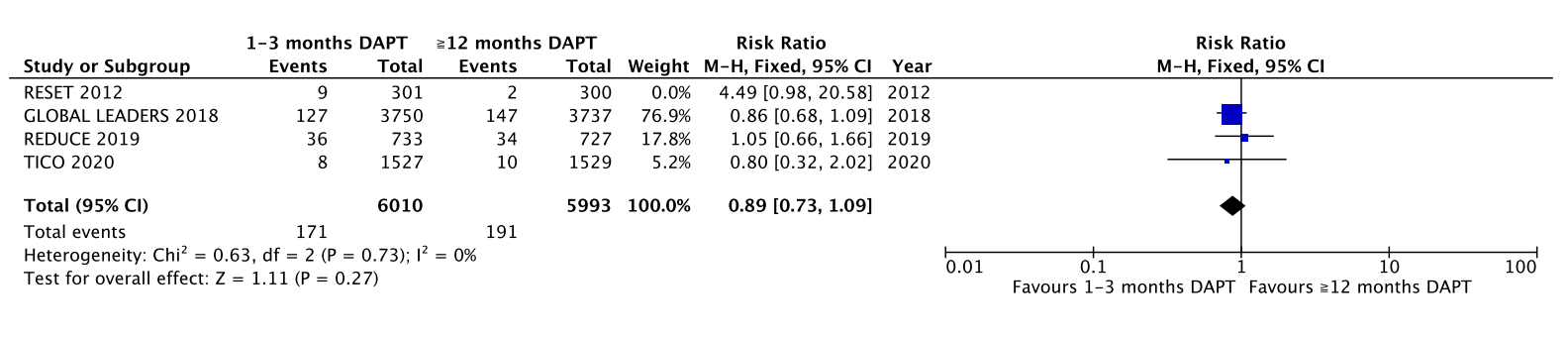


**Supplemental Figure S3.** The forest plot of target vessel revascularization after excluded the RESET trial.


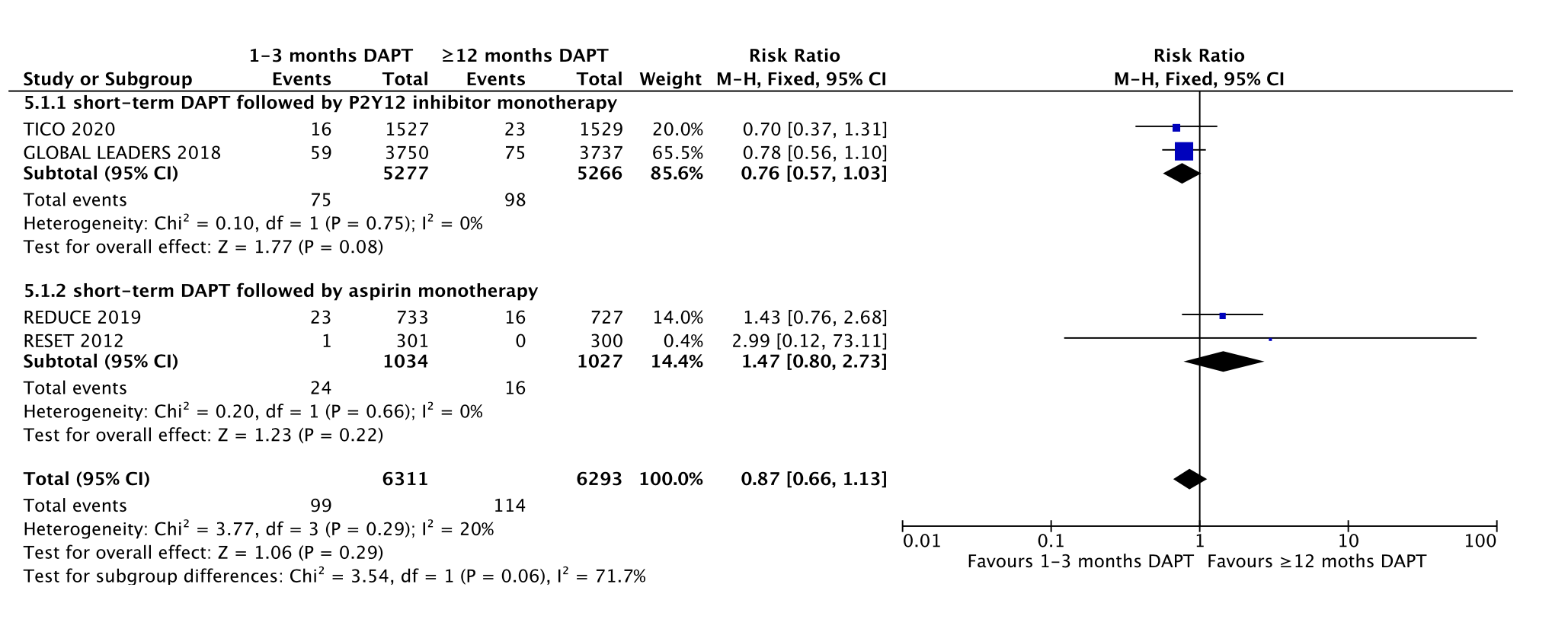


**Supplemental Figure S4.** The forest plot of subgroup analysis for all-cause death.


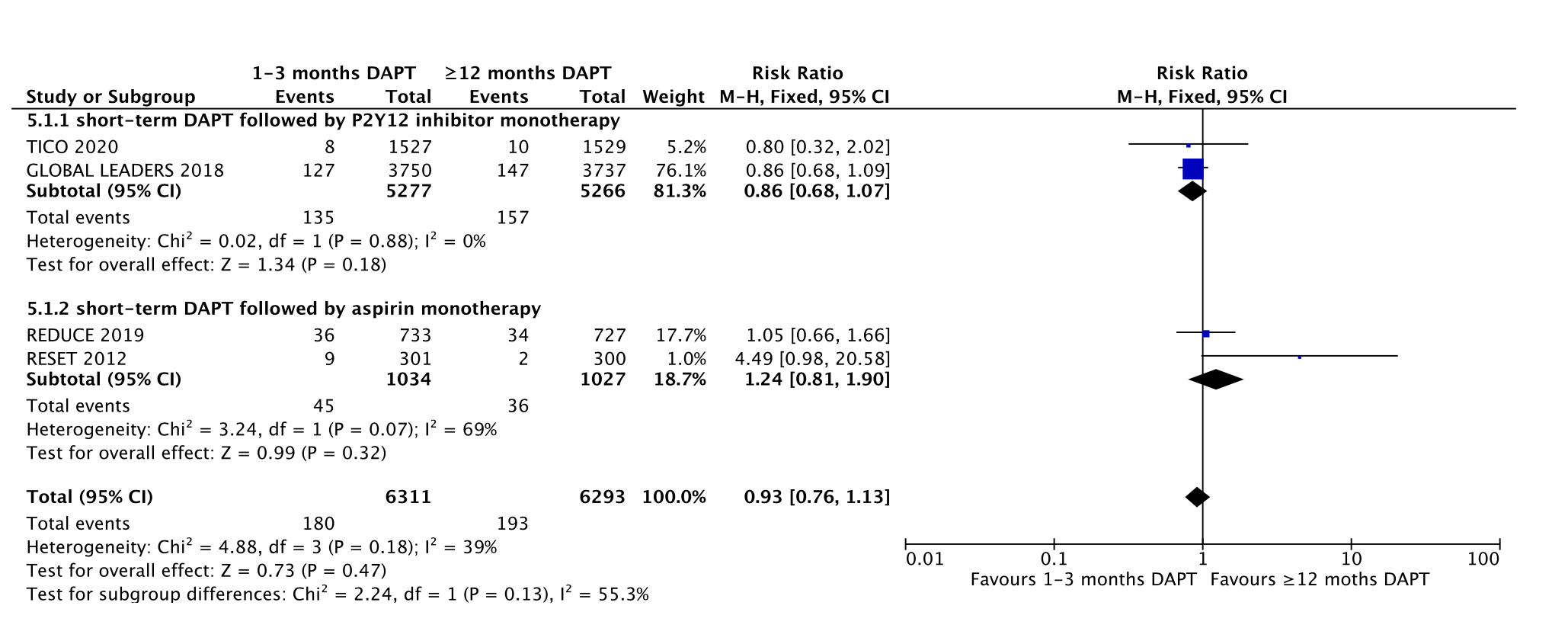


**Supplemental Figure S5.** The forest plot of subgroup analysis for stent thrombosis.

A B


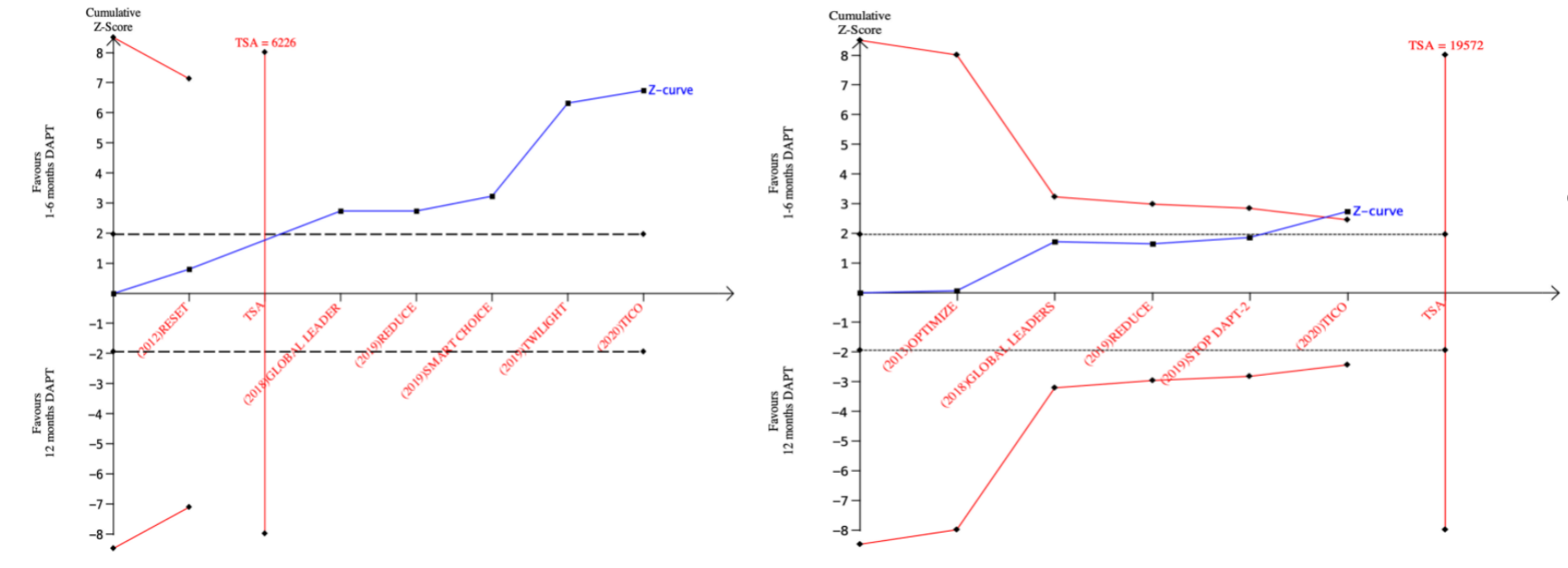


C D


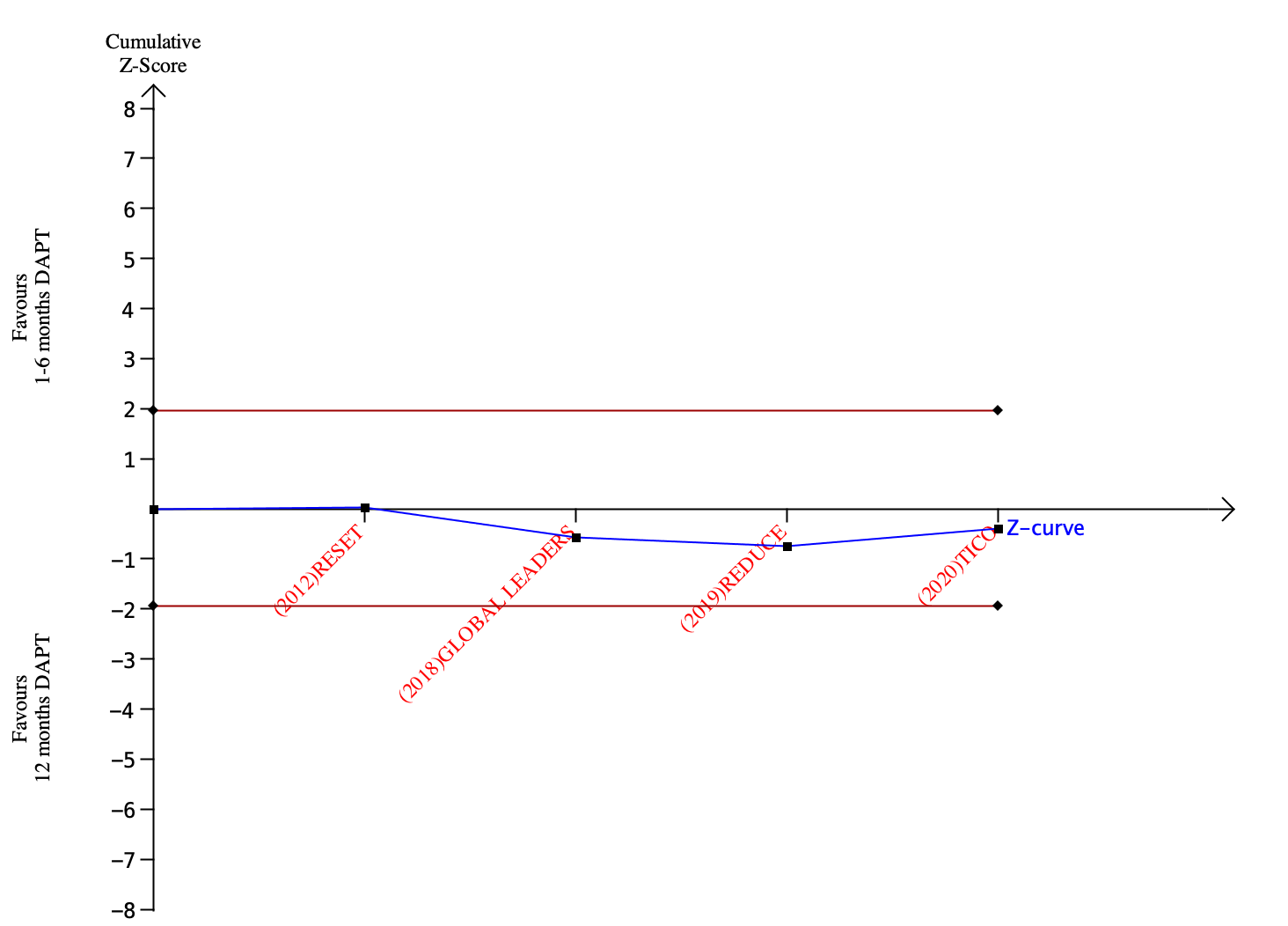

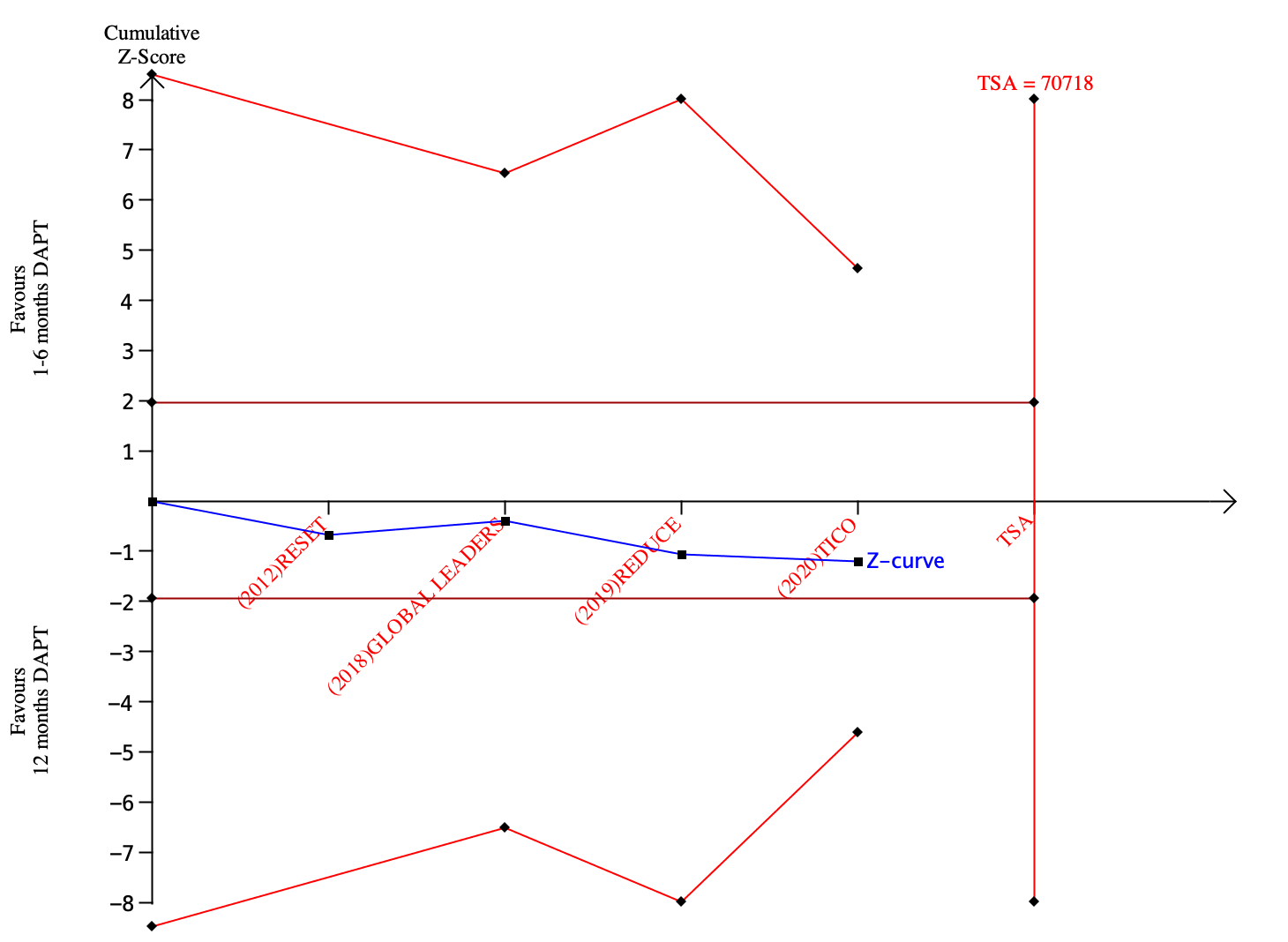


E F


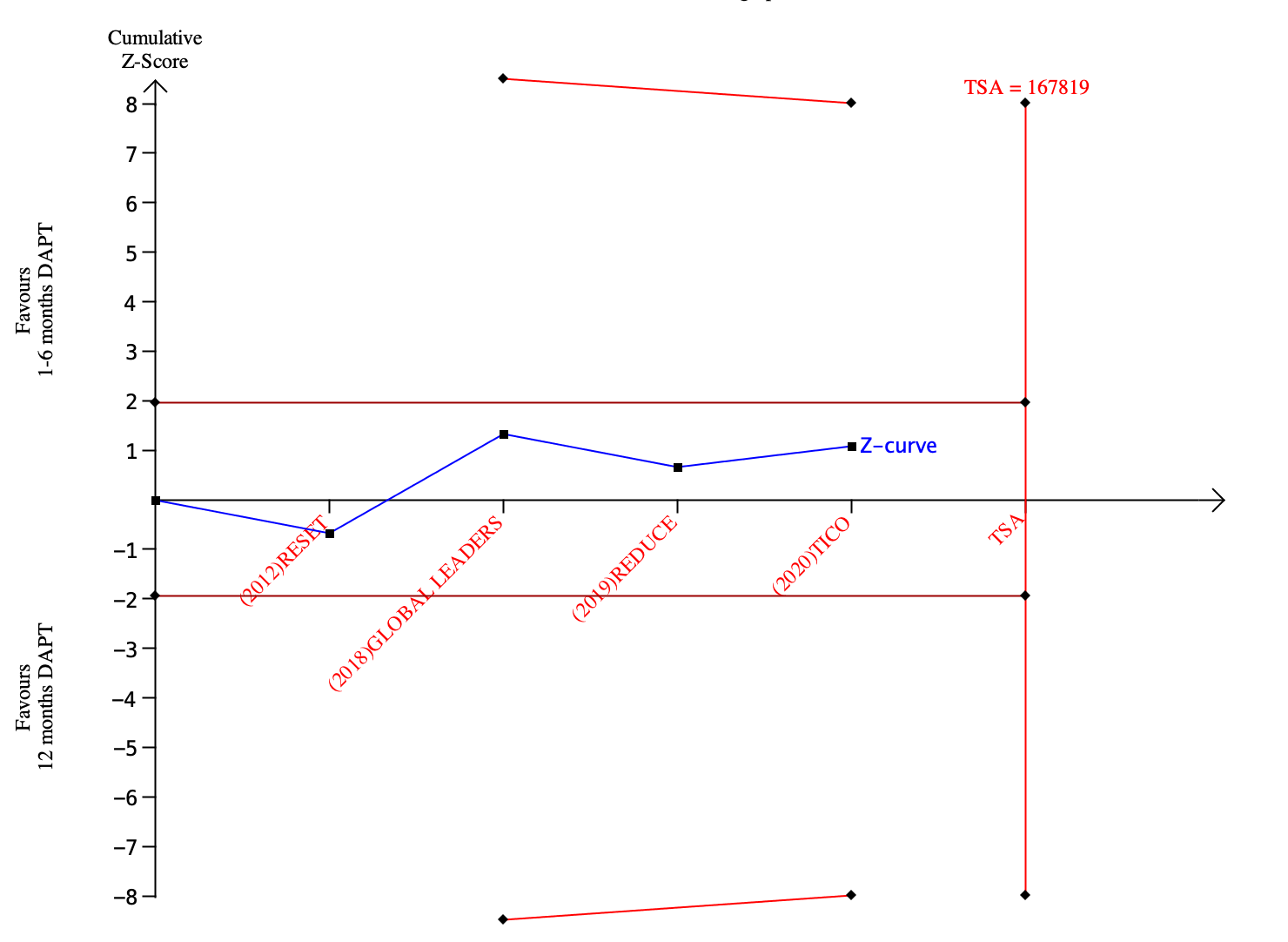

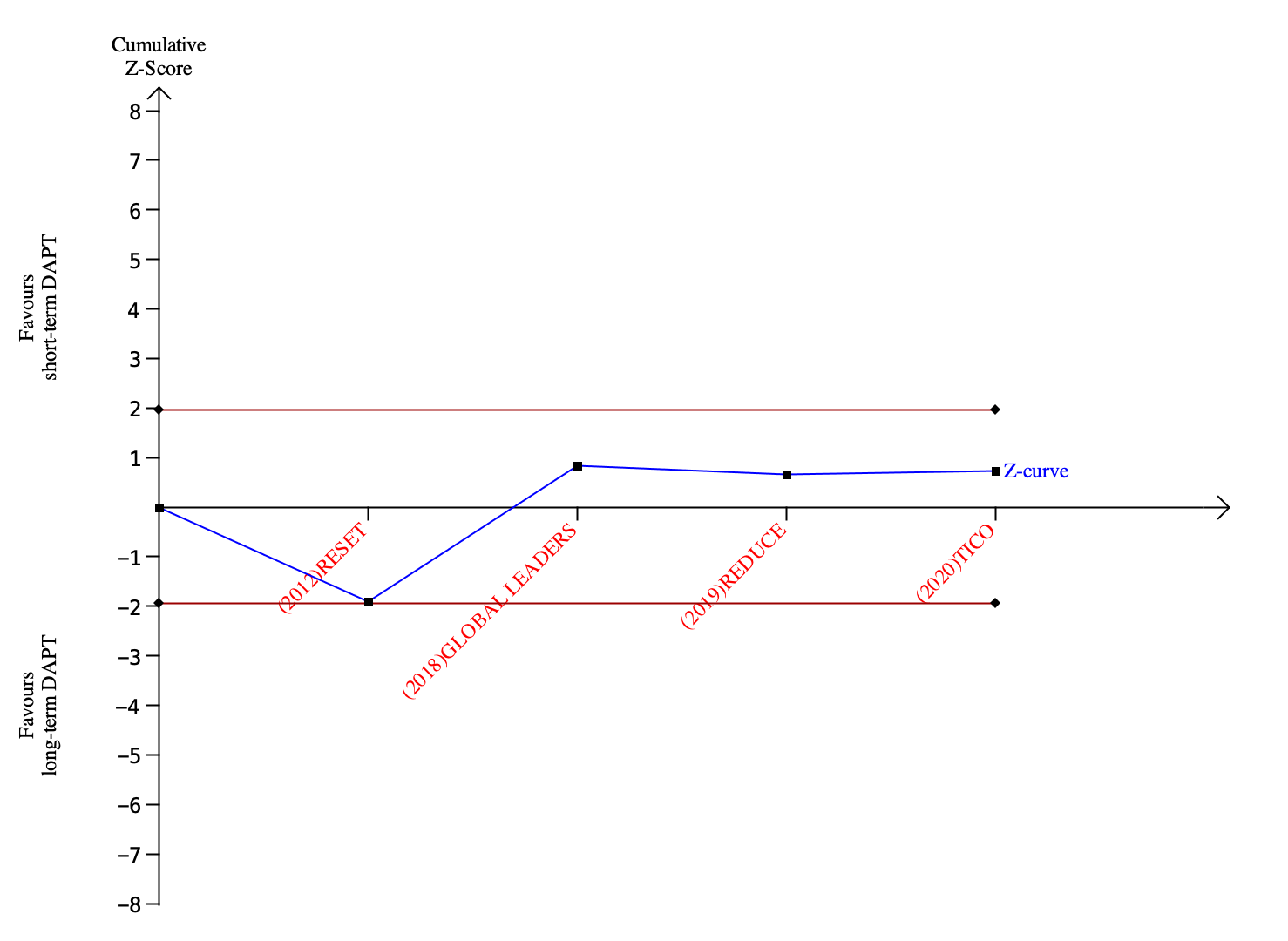


**Supplemental Figure S6.** The results of TSA between short-term and long-term DAPT groups. (A) major bleeding, (B) NACCE, (C) myocardial infarction, (D) stent thrombosis, (E) all-cause death, (F) target vessel revascularization.


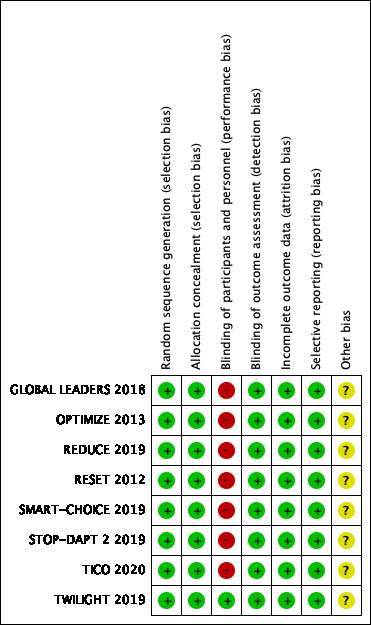


**Supplemental Figure S7**. Bias risk assessment of the studies.


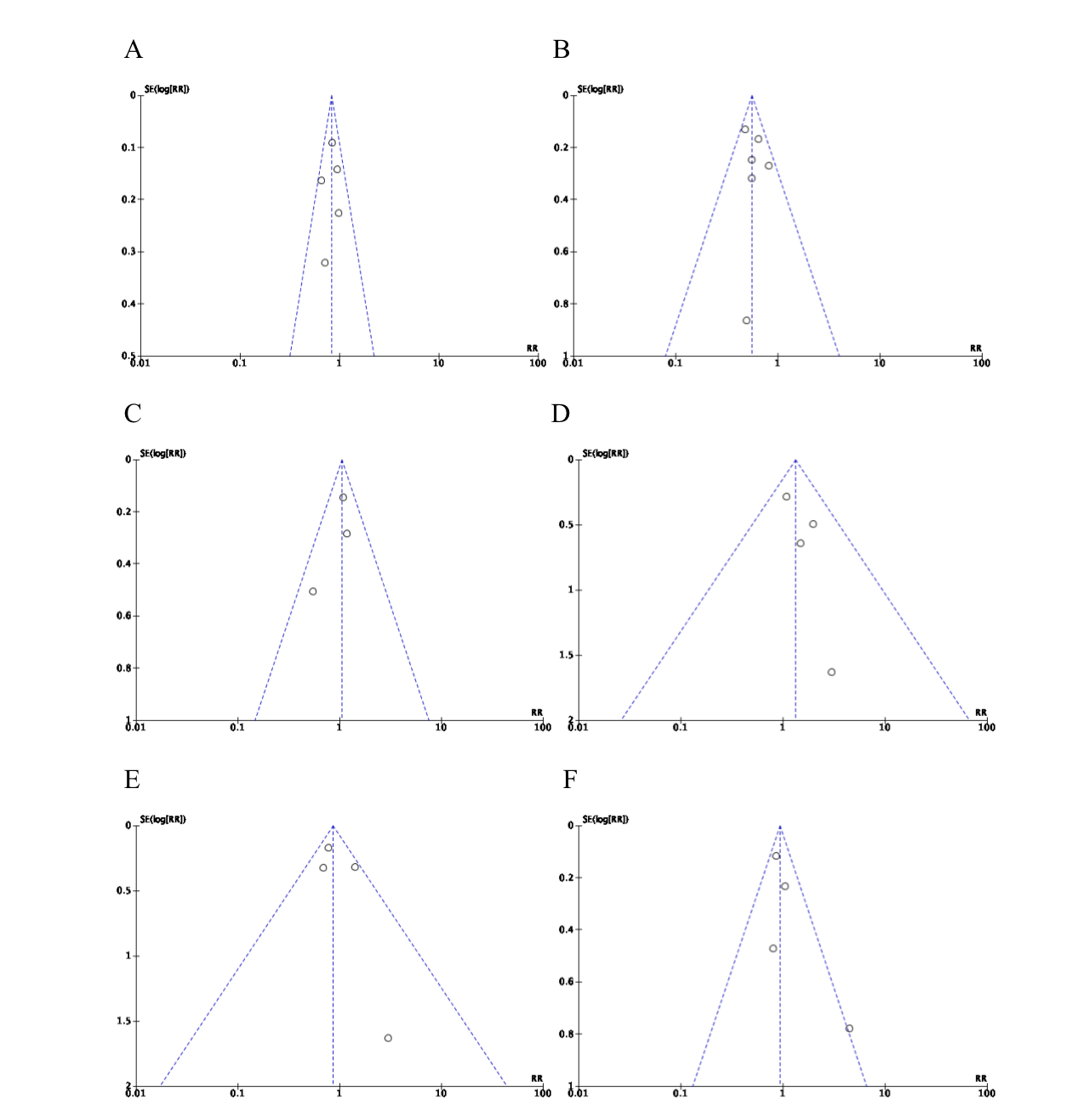


**Supplemental Figure S8.** The funnel plots of each outcome. (A) NACCE, (B) major bleeding, (C) myocardial infraction, (D) stent thrombosis, (E) all-cause mortality, (F) target vessel revascularization.
